# SignalP 6.0 predicts all five types of signal peptides using protein language models

**DOI:** 10.1038/s41587-021-01156-3

**Published:** 2022-01-03

**Authors:** Felix Teufel, José Juan Almagro Armenteros, Alexander Rosenberg Johansen, Magnús Halldór Gíslason, Silas Irby Pihl, Konstantinos D. Tsirigos, Ole Winther, Søren Brunak, Gunnar von Heijne, Henrik Nielsen

**Affiliations:** 1 Section for Bioinformatics, Department of Health Technology, Technical University of Denmark, Kongens Lyngby, Denmark; 2 Department of Biosystems Science and Engineering, ETH Zurich, Basel, Switzerland; 3 Novo Nordisk Foundation Center for Protein Research, Faculty of Health and Medical Sciences, University of Copenhagen, Copenhagen, Denmark; 4 Department of Computer Science, Stanford University, Stanford, CA USA; 5 Center for Genomic Medicine, Rigshospitalet (Copenhagen University Hospital), Copenhagen, Denmark; 6 EMBL-EBI, Wellcome Genome Campus, Cambridge, UK; 7 Department of Biology, Bioinformatics Centre, University of Copenhagen, Copenhagen, Denmark; 8 Section for Cognitive Systems, Department of Applied Mathematics and Computer Science, Technical University of Denmark, Kongens Lyngby, Denmark; 9 Department of Biochemistry and Biophysics, Stockholm University, Stockholm, Sweden; 10 Science for Life Laboratory, Stockholm University, Solna, Sweden

**Keywords:** Proteolysis, Protein sequence analyses, Sequence annotation, Protein translocation

## Abstract

Signal peptides (SPs) are short amino acid sequences that control protein secretion and translocation in all living organisms. SPs can be predicted from sequence data, but existing algorithms are unable to detect all known types of SPs. We introduce SignalP 6.0, a machine learning model that detects all five SP types and is applicable to metagenomic data.

## Abstract (web summary)

A new version of SignalP predicts all types of signal peptides.

## Main

SPs are short N-terminal amino acid sequences that target proteins to the secretory (Sec) pathway in eukaryotes and for translocation across the plasma (inner) membrane in prokaryotes. As comprehensive experimental identification of SPs is impractical, computational prediction of SPs has high relevance to research in cell biology^1^. SP prediction tools enable identification of proteins that follow the general secretory or twin-arginine translocation (Tat) pathway and predict the position in the sequence where a signal peptidase (SPase) cleaves the SP^2,3^. SignalP 5.0 is able to predict Sec substrates cleaved by SPase I (Sec/SPI) or SPase II (Sec/SPII, prokaryotic lipoproteins) and Tat substrates cleaved by SPase I (Tat/SPI)^4^. However, due to a lack of annotated data, SignalP 5.0 is unable to detect Tat substrates cleaved by SPase II or Sec substrates processed by SPase III (prepilin peptidase, sometimes referred to as SPase IV^2^). Such Sec/SPIII SPs control the translocation of type IV pilin-like proteins, which play a key role in adhesion, motility and DNA uptake in prokaryotes^5^. Furthermore, SignalP 5.0 is agnostic regarding the SP structure, as it cannot define the subregions (the N-terminal n-region, the hydrophobic h-region, and the C-terminal c-region) that underlie the biological function of SPs.

Here, we present SignalP 6.0, based on protein language models (LMs)^6–9^ that use information from millions of unannotated protein sequences across all domains of life. LMs create semantic representations of proteins that capture their biological properties and structure. Using these protein representations, SignalP 6.0 can predict additional types of SPs that previous versions have been unable to detect while better extrapolating to both proteins distantly related to those used to create the model and metagenomic data of unknown origin. In addition, it is capable of identifying the subregions of SPs.

**Fig. 1:**
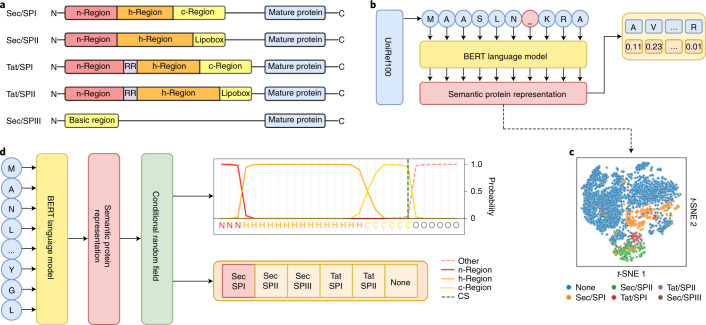
Modeling SP structure using protein LMs. **a**, Region structures of the five SP types. Twin arginine (RR)-translocated SPs feature a twin-arginine motif, while SPs cleaved by SPase II feature a C-terminal lipobox. Sec/SPIII SPs have no substructure. **b**, Protein LM training procedure. BERT learns protein features by predicting masked amino acids in sequences from UniRef100. **c**, *t*-Distributed stochastic neighbor embedding (*t*-SNE) projection of protein representations before prediction training. Different SP types form distinct clusters, separated from sequences without SPs. **d**, SignalP 6.0 architecture. An amino acid sequence is passed through the LM, and the resulting representation serves as input for the CRF, which predicts region probabilities at each position and the SP type. CS, cleavage site.

We compiled a comprehensive dataset of protein sequences that are known to harbor SPs, containing 3,352 Sec/SPI, 2,261 Sec/SPII, 113 Sec/SPIII, 595 Tat/SPI, 36 Tat/SPII, 16,421 intracellular sequences and 2,615 transmembrane sequences (Methods). Moreover, we defined region-labeling rules according to known properties of the SP types (Fig. 1a and Methods). We applied threefold nested cross-validation to train and evaluate the model (Methods and Supplementary Note 1). In our data-partitioning procedure, we ensured that homologous sequences were placed in the same partition to accurately measure the model’s performance on unseen sequences.

For previous predictors, the SP types Sec/SPIII and Tat/SPII were omitted due to a lack of annotated samples, which makes learning their defining features challenging for models^4^. Notably, this lack does not correspond to prevalence in nature, as these types exist throughout most organisms present in the databases^10,11^. In addition, the available annotated sequences do not cover the full diversity encountered in nature, as they are biased towards well-studied organisms. Furthermore, existing predictors require data for which the organism of origin is known, as this allows the predictors to explicitly account for known differences in SP structure among Eukarya, Archaea and Gram-positive and Gram-negative bacteria.

Protein LMs have been shown to improve performance on problems with limited annotated data^12^. Moreover, LM protein representations directly capture the evolutionary context of a sequence^6,8^. We hypothesized that when using an LM, we would (1) obtain better performance on SP types with limited data availability, (2) achieve better generalization to sequences that are distantly related to training sequences and (3) enable the prediction of sequences for which the species of origin is unknown. We opted for the bidirectional encoder representations from transformers (BERT) protein LM, which is available in ProtTrans^6,7^ and was trained on UniRef100 (ref. ^13^) (Fig. 1b). The LM was subsequently optimized on our dataset to predict SPs. We found that even before optimization, the LM captured the presence of SPs in its protein representations (Fig. 1c). We combined the LM with a conditional random field (CRF) probabilistic model^14^ to predict the SP region at each sequence position together with the SP type, yielding the SignalP 6.0 architecture (Fig. 1d).

**Fig. 2:**
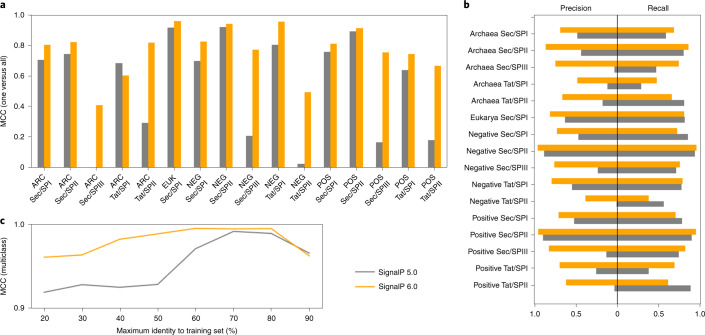
SignalP 6.0 shows strong performance on all types and organism groups. **a**, SP detection performance (ARC, Archaea; EUK, Eukarya; NEG, Gram-negative bacteria; POS, Gram-positive bacteria). SignalP 6.0 substantially improves performance on underrepresented types. **b**, CS prediction performance. SignalP 6.0 has improved precision for all categories. **c**, Dependence of performance on identity to sequences in the training data. At sequence identities lower than 60%, SignalP 6.0 outperforms SignalP 5.0.

As the baseline for evaluation, we retrained SignalP 5.0 on our new dataset. We measure performance for each SP type separately per organism group (Archaea, Eukarya, Gram-positive and Gram-negative bacteria), reporting the Matthews correlation coefficient (MCC) for correctly detecting the SP type among both non-SP and other types of sequences as negative samples. For all categories except Tat/SPI in Archaea, SignalP 6.0 showed improved performance. Detection performance improved substantially, especially for the two underrepresented types, Sec/SPIII and Tat/SPII (Fig. 2a and Supplementary Fig. 1), whereas the performance of SignalP 5.0 remained too low to make it practically useful. This confirms the importance of LMs for low-data problems, making SignalP 6.0 a model capable of simultaneously detecting all five types of SPs. In addition, we found substantial precision gains for predicting cleavage sites (CSs) (Fig. 2b).

We further benchmarked SignalP 6.0 against other publicly available predictors. In some cases, specialized predictors show stronger performance on the specific tasks they were optimized for (Supplementary Figs. 2 and 3 and Supplementary Tables 1–6). However, none of these predictors are capable of detecting all SP types, and the results are further biased, as they cannot be evaluated in a cross-validated setup.

When predicting a set of test sequences grouped by identity to any sequence in the training data, we find that detection performance at high sequence identities remained comparable. However, at identities lower than 60%, SignalP 6.0 outperformed SignalP 5.0, showing better generalization to proteins distantly related to those present in the training data (Fig. 2c).

Most SP predictors require knowledge of a sequence’s organism group of origin for optimal performance^4,15,16^. SignalP 6.0 does not show reduced performance if this information is removed, indicating that the evolutionary context, as encoded in the LM representation, already captures the organism group (Supplementary Fig. 4). Ultimately, this makes SignalP 6.0 a multiclass SP prediction tool that is applicable to sequences of unknown origin, as is typically the case in metagenomic and metatranscriptomic assemblies. However, SignalP 6.0 still relies on start codons being correctly identified before application. For context, 1.7% of UniProt release 2021_02 entries (i.e., 3.5 million sequences) have no organism specified.

SPs are traditionally described as consisting of three regions. We benchmarked our region identification by comparing the properties of predicted regions to known properties^17^, with predictions matching all expected properties (Supplementary Note 2 and Supplementary Fig. 5). We additionally predicted a library of synthetic SPs that are either functional or nonfunctional in *Bacillus subtilis*^18^, revealing significant differences in the two groups’ regions that could not be identified before by traditional sequence analysis (Supplementary Fig. 6 and Supplementary Table 7).

This study presents SignalP 6.0, a machine learning model covering all five known types of SPs that accurately predicts both sequences of unknown origin and evolutionarily distant proteins. Through the use of protein LMs, SignalP 6.0 is able to predict types with very limited training data available. By making the full spectrum of SPs accessible, the model allows us to further improve our understanding of protein translocation throughout evolution (Supplementary Note 3 and Supplementary Tables 8 and 9). In addition, identification of SP regions opens up new avenues into researching the defining properties that govern SP functionality. Given the potential of SPs as drug targets^19^ and their emerging role in synthetic biology^18^, investigating SPs and their properties at scale may lead to further advances in these fields.

## Methods

### Sequence data

The dataset for SignalP 6.0 was obtained by extending the data published with SignalP 5.0 (ref. ^4^). For all classes that were already part of the original data (Sec/SPI, Sec/SPII, Tat/SPI and soluble and transmembrane proteins), we added sequences that had become available in the respective source databases (UniProt^20^ and Prosite^21^ for SPs and UniProt and TOPDB^22^ for soluble and transmembrane proteins) from 2018 until 7 November 2020, following the original selection criteria.

Tat/SPII sequences were identified using the combination of Prosite profiles PS51318 (Tat motif) and PS51257 (lipoprotein motif). By default, PS51318 is subject to postprocessing that prevents both profiles from matching the same sequence. As there is experimental evidence for the existence of Tat-translocated lipoproteins^10,11^, we considered this postprocessing rule to be biologically implausible. We disabled it manually in ScanProsite^23^ and scanned all prokaryotic sequences in Swiss-Prot, yielding a total of 25 sequences in which both profiles matched. Additional Tat/SPII sequences were found by training a simplified SignalP 6.0 model to discriminate SPII from non-SP sequences. We used this model to predict all Tat/SPI sequences in the training data, as we assumed that PS51257 is not sensitive enough to find all lipoproteins. We investigated the resulting hits in UniProt for supporting evidence that the proteins were true lipoproteins, yielding 12 sequences that we relabeled Tat/SPII from Tat/SPI. One additional sequence with manual evidence was found in the TatLipo 1.03 training data^10^. For Sec/SPIII sequences, we used Prosite pattern PS00409 for bacteria and Pfam^24^ family PF04021 for Archaea, yielding 103 and 10 sequences, respectively.

We improved the organism type classification of sequences by defining Gram-negative and Gram-positive bacteria more stringently, as we found that for edge cases such as Thermotogae, in which both gram stains can be observed^25^, the classification in SignalP 5.0 was unclear. We redefined Gram positive as all bacterial phyla that have a single membrane (monoderm): Actinobacteria, Firmicutes, Tenericutes, Thermotogae, Chloroflexi and Saccharibacteria. All remaining phyla have a double membrane (diderm) and were classified as Gram negative.

We followed the methodology introduced by Gíslason et al.^26^ for homology partitioning of the dataset into three partitions at 30% sequence identity. In brief, it achieves partitioning by computing the pairwise global sequence identities of all sequences using the Needleman–Wunsch algorithm^27^, followed by single-linkage clustering. The resulting clusters were grouped together into the desired number of partitions. If there were sequences in a partition that had pairwise identities to any sequence in another partition that were higher than the defined threshold, then those sequences were iteratively removed until the maximum sequence identity criterion was fulfilled. We performed the partitioning procedure separately for each SP type and the negative set, yielding three partitions for each of the six classes. The algorithm was further constrained to ensure that each generated partition was balanced for the four organism groups. We concatenated the resulting 3 × 6 partitions to yield the three final partitions for cross-validation, thereby ensuring that both the SP types and the organism groups were equally represented across partitions.

The CD-HIT clustering method^28^ that was employed in SignalP 5.0 enforces the homology threshold for cluster centers. However, as the training set was not homology reduced but rather homology clustered, other data points can have a homology overlap notably above the chosen threshold of 20% (Supplementary Fig. 7). When using the partitioning method of Gíslason et al., which strictly enforces the defined threshold, 20% maximum identity was impossible to achieve. Even at the relaxed threshold of 30%, the procedure resulted in the removal of a substantial part of the dataset to achieve separation in three partitions (Supplementary Table 10).

For benchmarking, we reused the benchmark set of SignalP 5.0, from which we excluded all sequences that were removed in the homology partitioning procedure of the new dataset. For sequences that were reclassified (to Gram positive or Tat/SPII), we changed the label accordingly (Supplementary Table 11).

For the synthetic SP dataset, we used the data reported by Wu et al.^18^. We gathered all synthetic SP-mature protein pairs that were experimentally characterized, yielding 57 nonfunctional and 52 functional sequences. For the region analysis, we only considered sequences predicted as Sec/SPI SPs by SignalP 6.0, reducing the number of nonfunctional sequences to 55.

Reference proteomes and proteins of unknown origin were obtained from UniProt release 2021_02. To identify sequences of unknown origin, we used taxonomy identifiers 48479 (environmental samples), 49928 (unclassified bacteria) and 2787823 (unclassified entries).

### Generation of SP region labels

We defined the task of learning SP regions as a multilabel classification problem at each sequence position. Multilabel differs from multiclass in the sense that more than one label can be true at a given position. This approach was motivated by the fact that there is no strict definition of region borders that is commonly agreed upon, making it impossible to establish ground-truth region labels for models to train on. We thus used the multilabel framework as a method for training with weak supervision, allowing us to use overlapping region labels during the learning phase that could be generated from the sequence data using rules. For inference, we did not make use of the multilabel framework, as we only predicted the single most probable label at each position using Viterbi decoding, yielding a single unambiguous solution.

We defined a set of three rules based on known properties of the n-, h-, and c-regions. The initial n-region must have a minimum length of two residues and the terminal c-region a minimum length of three residues. The most hydrophobic position, which is identified by sliding a seven-amino-acid window across the SP and computing the hydrophobicity using the Kyte–Doolittle scale^29^, belongs to the h-region. All positions between these six labeled positions are labeled as either both n and h or h and c, yielding multitag labels.

This procedure was adapted for different SP classes, with only Sec/SPI completely following it. For Tat SPs, the n–h border was identified using the twin-arginine motif. All positions before the motif were labeled n, followed by two dedicated labels for the motif, again followed by a single position labeled n. For SPII SPs, we did not label a c-region, as the C-terminal positions cannot be considered as such^30^. The last three positions were labeled as the lipobox, all positions before that as h only. For SPIII SPs, no region labels were generated within the SP.

### Modeling

SignalP 6.0 uses a pretrained protein LM to encode the amino acid sequence and a CRF^14^ decoder to predict the regions, CSs and sequence class labels. Specifically, we used the 30-layer BERT LM ^31^ that is available in ProtTrans^6^, which was pretrained on UniRef100 (ref. ^13^). We removed the last layer of the pretrained model and extended the pretrained embedding layer by four additional randomly initialized vectors to represent the tokens for the four organism group identifiers. We prepend the organism group identifier to each sequence *s* of length *T* and encode it. From the resulting sequence of hidden states, we trim the positions corresponding to the organism group token and the special sequence start and end tokens used by BERT (CLS and SEP) to obtain a sequence of hidden states *h* of equal length as the original amino acid input *x*:

$$h = \mathrm{BERT}(x).$$

The hidden states serve as input for a linear-chain CRF. The CRF models the conditional probability of a sequence of states $y = y_1...y_t$ given a sequence of hidden states $h = {\bf{h}}_{\bf{1}}...{\bf{h}}_{\bf{t}}$ using the following factorization:

$$P(y|h) = \frac{1}{{Z(h)}}\mathop {\prod }\limits_{t = 1}^T {\mathrm{exp}}(\psi ({\bf{h}}_{\bf{t}}))\mathop {\prod }\limits_{t = 1}^{T - 1} {\mathrm{exp}}(\varphi _{y_t,y_{t + 1}}),$$

where *Z*(*h*) is the normalization constant of the modeled distribution; *φ* is the learnable transition matrix of the CRF with *C* × *C* parameters, with *C* being the number of states (labels) modeled by the CRF; and *ψ* is a learnable linear transformation that maps from the dimension of the hidden state h to the number of CRF states *C*, yielding the emissions for the CRF:

$$\psi (h_t) = W_\psi {\bf{h}}_{\bf{t}} + b_\psi.$$

For each class of SP G, there are multiple possible CRF states, corresponding to the defined regions of the SP class. We constrained the transitions in *φ* to ensure that regions are predicted in the correct order, leading to the possible state sequences depicted in Supplementary Fig. 8.

For inference, we compute both the most probable state sequence (using Viterbi decoding) and the marginal probabilities at all sequence positions (using the forward-backward algorithm). The most probable state sequence is used to predict the CS, which is inferred from the last predicted SP state as indicated in Supplementary Fig. 8.

As each SP consists of multiple regions, multiple states of C belong to a single global sequence class G. To predict the global class probabilities, we sum the marginal probabilities of all states that belong to a given class and divide the sum by the sequence length. This transforms a matrix of probabilities of shape C × T to a G × 1 vector of global class probabilities:

$$p(G_i|x) = \frac{1}{T}\mathop {\prod }\limits_{t = 1}^T \mathop {\sum }\limits_{C \in G_i} p(y_{Ct}|x).$$

### Training

For training, we minimize the negative log likelihood of the CRF. As we can have multiple true labels *y*_*t*_ at a given position, we use an extension of the equation known as multitag CRF. Multiple labels are handled by summing over the set of true labels *M*_*t*_ at each position:

$$- {\mathrm{log}}\left( {P\left( {y|h} \right)} \right) = {\mathrm{log}}\left( {Z\left( h \right)} \right) - {\mathrm{log}}\left( {\mathrm{exp}\left( {\mathop {\sum }\limits_{t = 1}^T \mathop {\sum }\limits_{y_t \in M_t} \psi \left( {\bf{h}}_{\bf{t}} \right) + \varphi \left( {y_t,y_{t - 1}} \right)} \right)} \right).$$

As we designed our region labels to be overlapping, the model is free to distribute its probability mass in any ratio between the correct labels at a given position. There are thus multiple solutions for the specific borders of n-, h- and c- regions that yield the same negative log likelihood but are not equally biologically plausible. For instance, the model could learn a solution where it uniformly predicts an n-region of length 2 in all SPs, irrespective of the actual sequence. We employ regularization to promote the finding of biologically plausible solutions. Our regularization is based on the fact that the three SP regions have divergent amino acid compositions, which we can quantify by computing the cosine similarity between the amino acid distributions.

The most obvious approach would be to compute the amino acid distribution of each region based on the region borders inferred from the predicted most probable path of the sequence. This, however, cannot be used for regularization, as we require the term to be differentiable, which our Viterbi decoding implementation is not. We therefore based our regularization term on the marginal probabilities of the CRF computed by the forward-backward algorithm, which are used to compute a score for each amino acid for each region, approximating the discrete amino acid distributions.

For each region $r \in \left\{ {n,h,c} \right\}$, we sum the marginal probabilities of all CRF states *c* belonging to region *r* at position *t*, yielding *s*_*t*,*r*_. We sum *s*_*t*,*r*_ of all positions *t* of the sequence that have amino acid *a*, yielding the elements of the score vector **score**_***r***_ for each region. We compute the cosine similarity between the normalized score vectors of *n* and *h* and *h* and *c*:

$$\begin{array}{l}s_{t,r} = \mathop {\sum }\limits_{c \in r} p\left( {y_{t,c}|x} \right)\\ {\mathrm{score}}_{a,r} = \mathop {\sum }\limits_{t \in I} s_{t,r}\\ I = \left\{ {t \in T|x_t = a} \right\}\\ {{\bf{score}}_{\bf{r}}}^\prime = {\bf{score}}_{\bf{r}}/\mathop {\sum }\limits_{a = 1}^A {{\mathrm{score}}_{{a,r}}}\end{array}.$$

We perform this operation for each sequence. Sequences for which a region does not exist (for example no c-region in Sec/SPII) are ignored for the respective similarity. The mean over all sequences for both similarities, multiplied by a factor *α*, was added to the loss. We observed that for about half the random seeds we tested, training runs with regularization enabled converged to a n-region length of 2 after one epoch. This is a degenerate solution, as this causes the n-region amino acid distribution to be nonzero at a single position, yielding low similarity scores while being biologically implausible (a length of 2 is expected as the minimum, not the average over all sequences). Such runs were stopped and discarded after one epoch.

The model was trained end-to-end, including all layers of BERT for 15 epochs, using Adamax as the optimizer and a slanted triangular learning rate. We applied dropout on the hidden state outputs of BERT to avoid overfitting. Hyperparameters were optimized using SigOpt (https://app.sigopt.com/docs/intro/overview). We employed threefold nested cross-validation (outer loop is threefold and inner loop is twofold), yielding a total of 3 × 2 models for evaluation.

### Evaluation and benchmarking

For comparability, we employed the same metrics that were used in SignalP 5.0. SP detection performance was measured using the MCC^32^. We computed the MCC twice, once with the negative set only consisting of transmembrane and soluble proteins (MCC1) and once with it additionally including sequences of all other SP types (MCC2). Most of the competing single-class predictors considered for benchmarking are optimized for detecting their respective SP type in a dataset of true and non-SP (soluble and transmembrane) sequences; thus, MCC1 best captures their performance on the task they were designed for. MCC2, on the other hand, includes the more challenging task of discriminating between SP types, which is difficult for single-class predictors because of the structural similarity of different SP types. MCC2 represents the performance in most real-world applications, as the presence of a specific SP type usually cannot be ruled out a priori in a set of unknown protein sequences. For CS prediction, we computed the precision and recall. Precision was defined as the fraction of correct CS predictions over the number of predicted CSs, and recall was defined as the fraction of correct CS predictions over the number of true CSs. In both cases, a CS was only considered correct if it was predicted in the correct SP class (e.g., when the model predicts a CS in a Sec/SPI sequence but predicts Sec/SPII as the sequence label, then the sample is considered ‘no CS predicted’). To account for possible uncertainty of the CSs in the training data labels, we additionally report these metrics with tolerance windows of one, two and three residues left and right of the true CS (Supplementary Tables 2, 4 and 6).

For the predicted SP regions, in the absence of true labels, no quantitative performance metrics could be established. To still be able to assess the quality of the predictions, we compared the properties of predicted regions with characteristics of regions that are described in the literature. We followed the review by Owji et al.^17^ as a guideline to identify region characteristics. Specifically, we evaluated the length, hydrophobicity and charge of each predicted region. Hydrophobicities were computed using the Kyte–Doolittle scale^29^, and charges were computed by summing the net charges at pH 7 of all residues. The net charge computation differed between the groups, as in Eukarya and Archaea the N-terminal methionine is not formylated^33^, thus contributing an additional positive charge to the n-region by its amino group.

We benchmarked our model against the state-of-the-art model SignalP 5.0, which was reimplemented in PyTorch. Hyperparameter optimization on the new dataset was performed using SigOpt. We also repeated the benchmarking experiment of SignalP 5.0 for all predictors using the adapted benchmark set. We could not add Signal-3L 3.0^16^ to the experiment, as the implementation that is available does not allow for processing of more than one sequence at a time, rendering benchmarking intractable. Notably, predictions for all methods except for SignalP 5.0 and SignalP 6.0 were obtained from their publicly available web services, resulting in potential performance overestimation due to the lack of homology partitioning. In addition, performance overestimation is still present for the published version of SignalP 5.0 (named “SignalP 5.0 original” in Supplementary Tables 1–6 and Supplementary Figs. 1 and 2) due to insufficient homology partitioning of its training data by CD-HIT. We thus excluded its values from determining the best-performing tools in the benchmark.

To assess the effect of sequence identity to training sequences on performance, we used the set of sequences that were removed by the partitioning procedure. We predicted all sequences in the removed set and binned the sequences according to the maximum sequence identity to any sequence in the training set. We did this for all six cross-validated models and pooled the resulting binned predictions. For each bin, we computed the multiclass MCC as defined by Gorodkin^34^.

### Reporting Summary

Further information on research design is available in the Nature Research Reporting Summary linked to this article

## Online content

Any methods, additional references, Nature Research reporting summaries, source data, extended data, supplementary information, acknowledgements, peer review information; details of author contributions and competing interests; and statements of data and code availability are available at 10.1038/s41587-021-01156-3.

## Supplementary information

Supplementary Information Supplementary Figures 1–8, Notes 1–3 and Tables 1–12

Reporting Summary

## Data Availability

The datasets used for training and testing SignalP 6.0 can be downloaded from https://services.healthtech.dtu.dk/service.php?SignalP-6.0. The investigated reference proteomes are available from UniProt at https://www.uniprot.org/proteomes. The dataset of synthetic SPs was extracted from the supplementary material of the original publication^18^ and is included in our GitHub repository.

## Code availability

SignalP 6.0 is available at https://services.healthtech.dtu.dk/service.php?SignalP-6.0. The web version of SignalP 6.0 is free for all users, while the standalone Python package is free for academic users (and can be provided upon request) but is licensed for a fee to commercial users. The model source code in PyTorch 1.7 is available at https://github.com/fteufel/signalp-6.0.
