# Supplementary material for: SignalP 6.0 predicts all five types of signal peptides using protein language models
Source: Nat Biotechnol. 2022 Jan 3;40(7):1023–5. doi: 10.1038/s41587-021-01156-3 (PMC9287161; doi:10.1038/s41587-021-01156-3)
Supplement: Supplementary file 1 — Supplementary Figures 1–8, Notes 1–3 and Tables 1–12 [file 41587_2021_1156_MOESM1_ESM.pdf]

nature portfolio

https://doi.org/10.1038/s41587-021-01156-3

Supplementary information

SignalP 6.0 predicts all five types of signal peptides using protein language models

In the format provided by the authors and unedited

Supplementary information for

SignalP 6.0 predicts all five types of signal peptides using protein language models

Authors: Felix Teufel[1,2], José Juan Almagro Armenteros[1,3], Alexander Rosenberg Johansen[4], Magnús Halldór Gíslason[5], Silas Irby Pihl[1], Konstantinos D. Tsirigos[6], Ole Winther[5,7,8], Søren Brunak[1,3], Gunnar von Heijne[9,10], Henrik Nielsen[1]*

Supplementary note 1: Model distillation

The SignalP 6.0 predictor is an ensemble model of all six models trained in nested cross-validation. Due to the computational demand of the BERT protein LM, this poses a challenge for prediction on systems with limited resources and results in long processing times for larger numbers of sequences. To still be able to offer prediction speeds comparable to previous versions of SignalP, we performed model distillation. For each sequence in the training set, we obtain the marginal probabilities predicted by the full ensemble model (termed *SignalP 6.0 - slow*). We instantiate a new SignalP 6.0 model from the pretrained LM, and train this model on the full training set, using the Kullback-Leibler divergence between its marginal probabilities and the ensemble model marginal probabilities as the loss. To mitigate class imbalance, we multiply the loss of each sequence with a weight inversely proportional to its type's prevalence in the training set. The model is trained until convergence, yielding *SignalP 6.0 - fast*.

By nature of the training objective, the distilled model is only trained to approximate the marginal probabilities and SP type probabilities of the full model (Supplementary Table 12). However, as the distilled model does not replicate the emissions, this can lead to discrepancies in the predicted Viterbi paths. We thus recommend using *SignalP 6.0 - slow* for applications where accurate region border predictions are of interest.

Supplementary note 2: Signal peptide region prediction results

Signal peptides are traditionally described as consisting of three distinct regions with different biochemical properties (1, 2). While it is possible to segment a SP sequence's regions by manual inspection of the sequence given sufficient domain knowledge, there is no clear set of rules on how to do so universally. Rather, expert annotators do so on a case-by-case basis to arrive at a probable solution. However, this approach is not scalable to proteome-level data, so far preventing detailed analysis of SP structure. As there is also no experimental technique known that identifies region borders accurately, SignalP 6.0 instead learns SP structure directly from unlabeled data in a weak supervision fashion (Online methods). This lack of labeled region data for training naturally also implies that no labeled data is available for measuring performance. Thus, we benchmark our region identification by comparing the properties of predicted regions to known properties from literature (2). Specifically, we evaluate the predicted lengths, hydrophobicities and net charges of n-, h-, and c-regions in all SP types. We find that the predictions of SignalP 6.0 match all expected properties. For n-regions, the model correctly recovers the average length and the differences between organism groups (17) (Supplementary Fig. 5). Predicted h-regions are less hydrophobic in Tat-translocated SPs than in Sec SPs, a property that is known to contribute to the selectivity of the pathways (3). While the c-region is generally uncharged, in Tat SPs it can contain positively charged residues to avoid recognition by the Sec system, a feature called the Sec avoidance motif (4). SignalP 6.0 accurately captures this property, with the majority of Tat/SPI c-regions having a net charge of 0 or +1. The model also predicts negatively charged Tat/SPI c-regions, hinting at negative charges also possibly being suitable to hinder recognition by the Sec pathway.

To further evaluate the region prediction capability, we predict a library of synthetic SPs that were found to be either functional or non-functional in Bacillus subtilis (5). In the original work, the authors did not find any discriminating properties between the two groups using traditional sequence analysis. Region predictions show a significant difference in n-region net charge ($P<1\times10\text{-}4$) and hydrophobicity ($P<1\times10\text{-}3$) between the groups (Supplementary Fig. 5, Supplementary Table 1), revealing possible factors that contribute to in vivo functionality.

Supplementary note 3: Proteome-wide predictions

To gain insight into the diversity of SP usage throughout evolution, we predicted all reference proteomes available in Uniprot (6) (Supplementary Tables 10-11). Predictions confirm exceptionally high Tat/SPII frequencies in Halobacteria, as they were previously reported (7), even though the training dataset only contains 3 such sequences. Moreover, our predictions also revealed bacterial species with high Tat/SPII and Sec/SPIII frequencies, highlighting the importance of including these two often overlooked modes of protein export when investigating proteomes. In general, the number of secretory proteins is predicted to be highly variable among different species. Among all 9,915 organisms present in the data, the only species without predicted SPs are bacterial endosymbionts, indicating that protein translocation and export are indispensable to free-living organisms.

It is possible to estimate the number of proteins with SPs in an organism using proteomic methods. Ivankov et al. (8) reported an estimated 11.5% of the E. coli proteome to have SPs. SignalP 6.0 predicts a total of 12.2 %, indicating that the model's genome-scale predictions yield estimates of plausible sizes.

When aggregating results on the organism group level, we observe further diversity in translocation strategies (Supplementary Table 11). Tat/SPII SPs are predicted to be the most frequent in Archaea, together with a low frequency of Sec/SPII, suggesting that lipoproteins are mostly exported via the Tat pathway. We further find that the overall frequency of secretory pathway proteins is the highest in Gram-negative bacteria. Notably, these results might be biased by the selection of organisms for which reference proteomes are available, as it is unclear how well they represent their complete organism group.

Supplementary references

1. von Heijne, G. The signal peptide. *J. Membr. Biol.* **115**, 195–201 (1990).

2. Owji, H., Nezafat, N., Negahdaripour, M., Hajiebrahimi, A. & Ghasemi, Y. A comprehensive review of signal peptides: Structure, roles, and applications. *Eur. J. Cell Biol.* **97**, 422–441 (2018).

3. Berks, B. C., Sargent, F. & Palmer, T. The Tat protein export pathway. *Mol. Microbiol.* **35**, 260–274 (2000).

4. Bogsch, E., Brink, S. & Robinson, C. Pathway specificity for a ΔpH-dependent precursor thylakoid lumen protein is governed by a 'sec-avoidance' motif in the transfer peptide and a 'sec-incompatible' mature protein. *EMBO J.* **16**, 3851–3859 (1997).

5. Wu, Z. *et al.* Signal Peptides Generated by Attention-Based Neural Networks. *ACS Synth. Biol.* **9**, 2154–2161 (2020).

6. The UniProt Consortium. UniProt: a worldwide hub of protein knowledge. *Nucleic Acids Res.* **47**, D506–D515 (2019).

7. Storf, S. *et al.* Mutational and Bioinformatic Analysis of Haloarchaeal Lipobox-Containing Proteins. *Archaea* vol. 2010 e410975 https://www.hindawi.com/journals/archaea/2010/410975/ (2010).

8. Ivankov, D. N. *et al.* How many signal peptides are there in bacteria? *Environ. Microbiol.* **15**, 983–990 (2013).

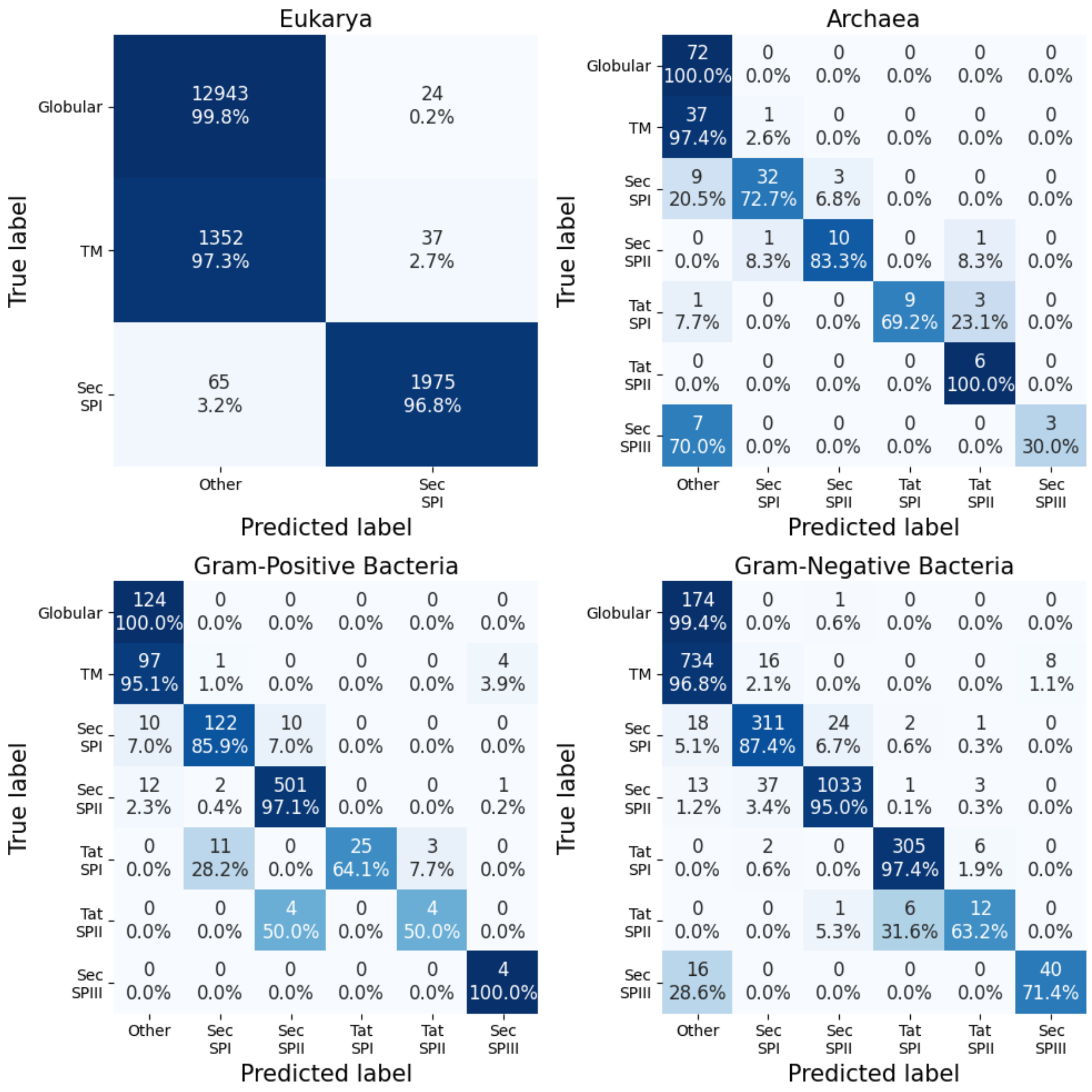

Supplementary Figure 1. Cross-validated confusion matrices of SignalP 6.0 for all organism groups. For the true labels, we divided the non-SP sequences into soluble proteins (Globular) and proteins with transmembrane regions in their first 70 residues (TM). Percentages are calculated row-wise to indicate the fraction of recovered true labels.

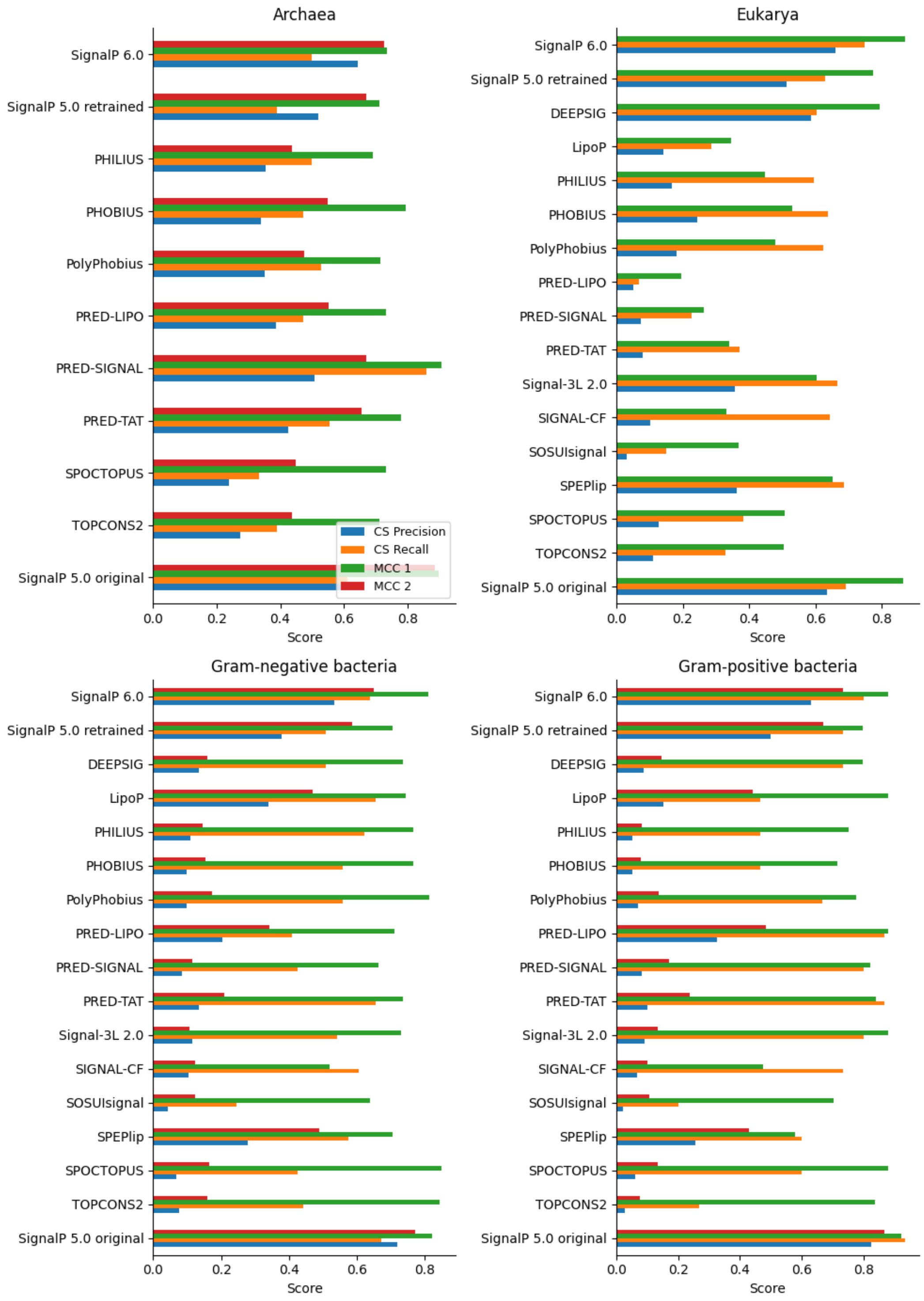

Supplementary Figure 2. Results of the benchmark experiment for Sec/SPI SPs. MCC1 refers to detection performance when the negative class consists of soluble and transmembrane proteins. For MCC2, the negative class additionally contains Sec/SPII, Tat/SPI and Tat/SPII SPs. CS recall and precision are calculated with a tolerance window size of 0. Note that the performance of SignalP 5.0 original is overestimated and only given for comparison.

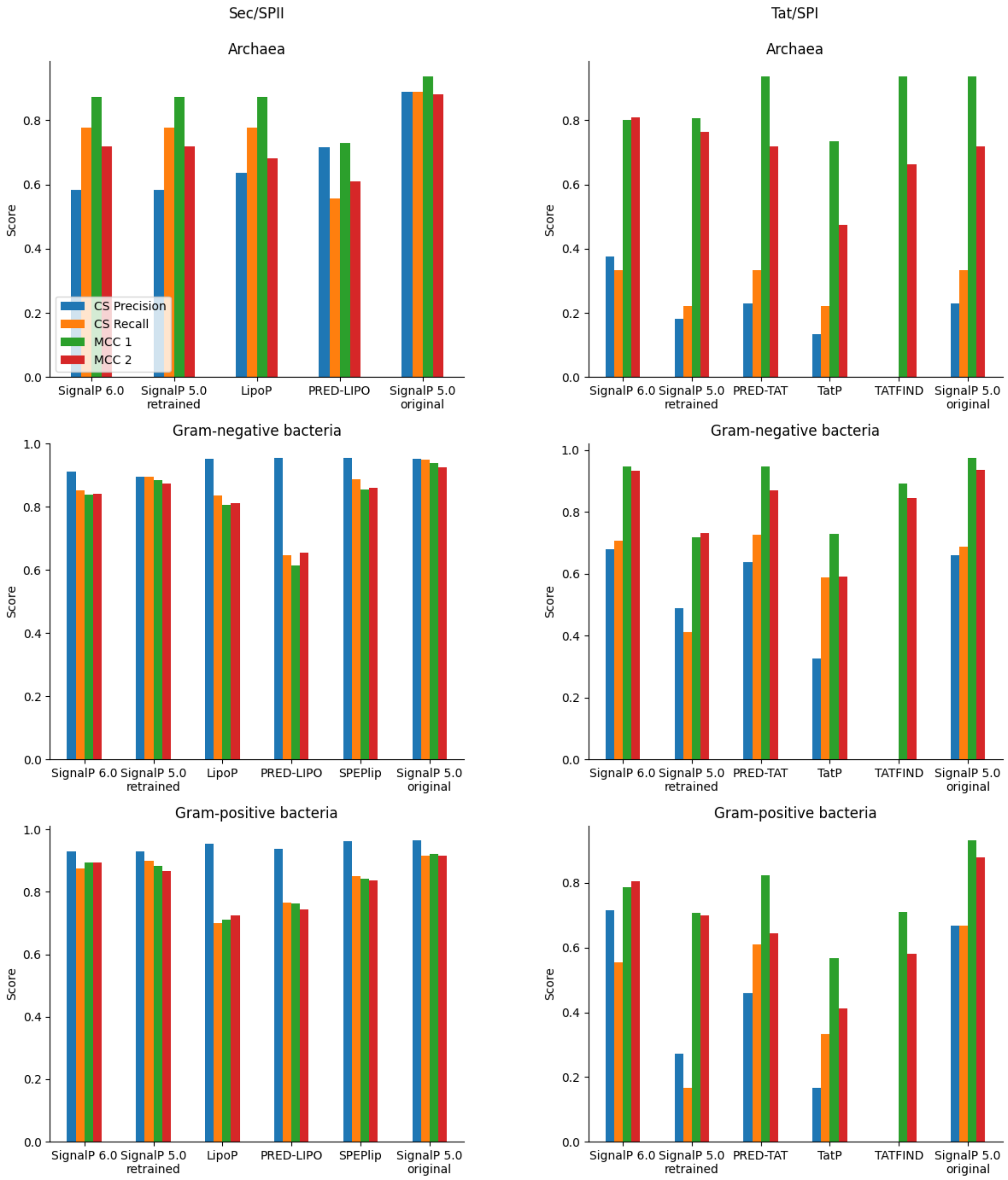

Supplementary Figure 3. Results of the benchmark experiment for Sec/SPII and Tat/SPI SPs. MCC1 refers to detection performance when the negative class consists of soluble and transmembrane proteins. For MCC2, the negative class additionally contains Sec/SPII, Tat/SPI and Tat/SPII SPs. CS recall and precision are calculated with a tolerance window size of 0. Note that the performance of SignalP 5.0 original is overestimated and only given for comparison.

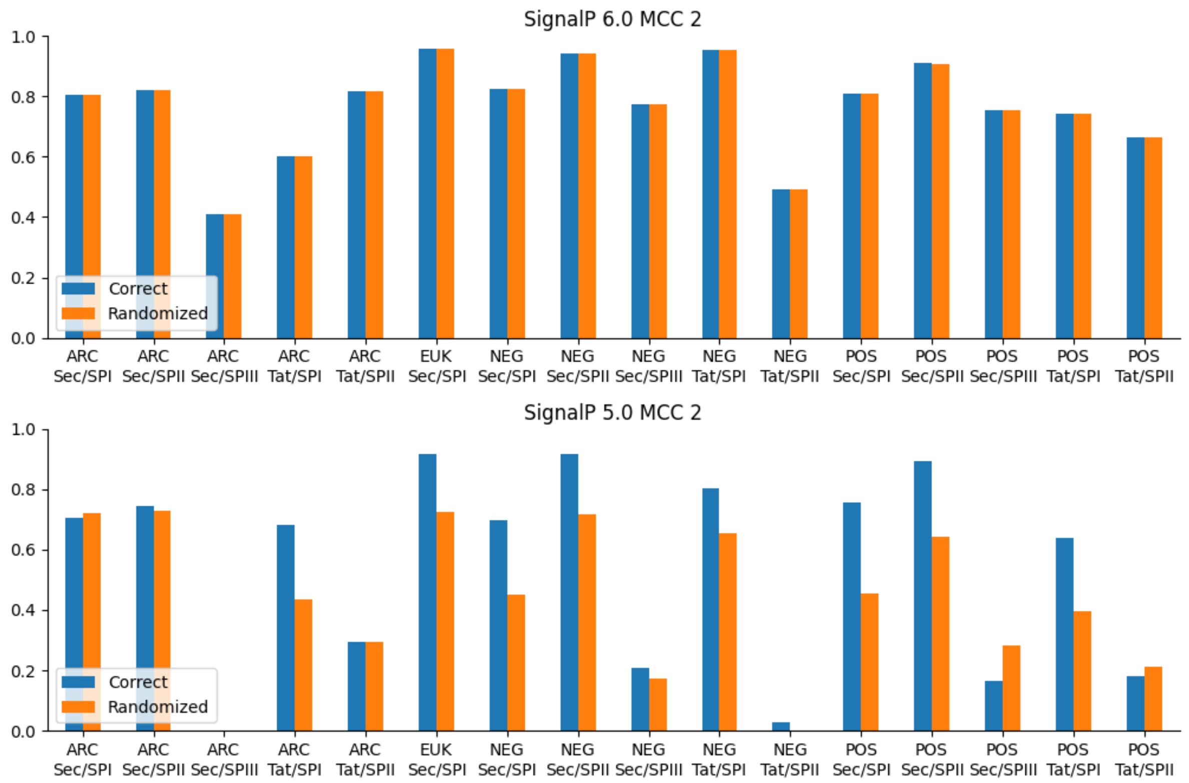

Supplementary Figure 4. Performance of SignalP 6.0 and SignalP 5.0 on data with correct and with randomized organism group identifiers.

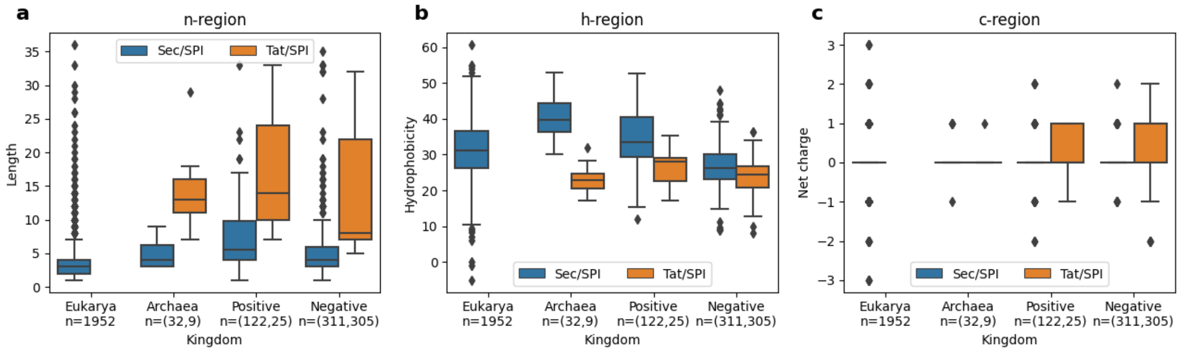

Supplementary Figure 5. Predicted regions recapitulate known properties. **(a)** n-region lengths. The expected average length of about 4 residues for Sec/SPI SPs is recovered, n-regions are correctly predicted to be the shortest in eukarya and the longest in Gram-positive bacteria. **(b)** Hydrophobicities of the h-regions of Sec/SPI and Tat/SPI SPs. Sec-translocated SPs are predicted to have a higher hydrophobicity. **(c)** Net charges of Sec/SPI and Tat/SPI c-regions. Sec c-regions are uncharged, Tat c-regions are charged. The number of sequences is given underneath each label. In case of paired boxplots, the first value refers to Sec/SPI, and the second to Tat/SPI.

Box plots bounds are the lower and upper quartile values, centre is the median. The lower whisker extends to the lowest observed value greater than the lower quartile minus 1.5 times the interquartile range (IQR) of the data, the upper whisker to the highest observed value lower than the upper quartile plus 1.5 times the IQR.

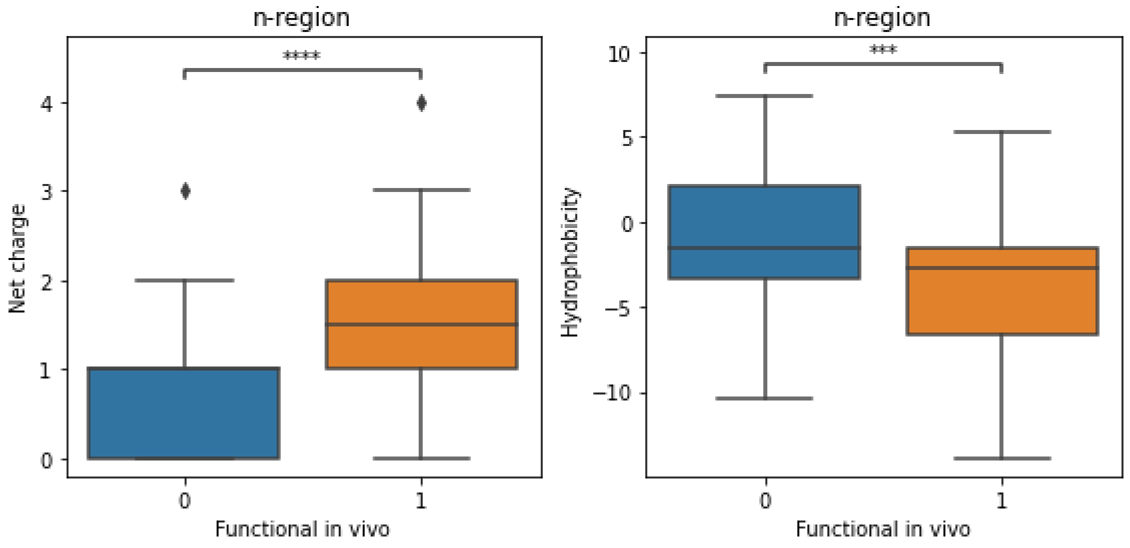

Supplementary Figure 6. Comparison of n-region properties of synthetic SPs. In *B. Subtilis*, group 0 was found to be nonfunctional (n=55 sequences), for group 1 protein secretion was observed (n=52 sequences) (****: $p<1\times10^{-4}$, ***: $p<1\times10^{-3}$, Welch's t-test, two-sided. Exact p-values: Net charge 1.821×10^{-5}, Hydrophobicity: 7.261×10^{-4}). Box plot parameters are the same as in Supplementary Figure 5.

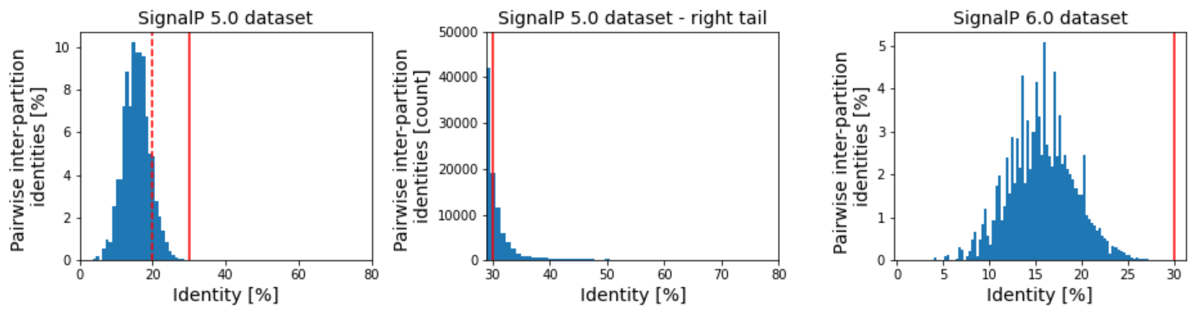

Supplementary Figure 7. Quality of homology partitioning. All pairwise inter-partition identities were computed using ggsearch36. The dashed line indicates the reported threshold for SignalP 5.0, the solid line the relaxed threshold of 30% used in this work.

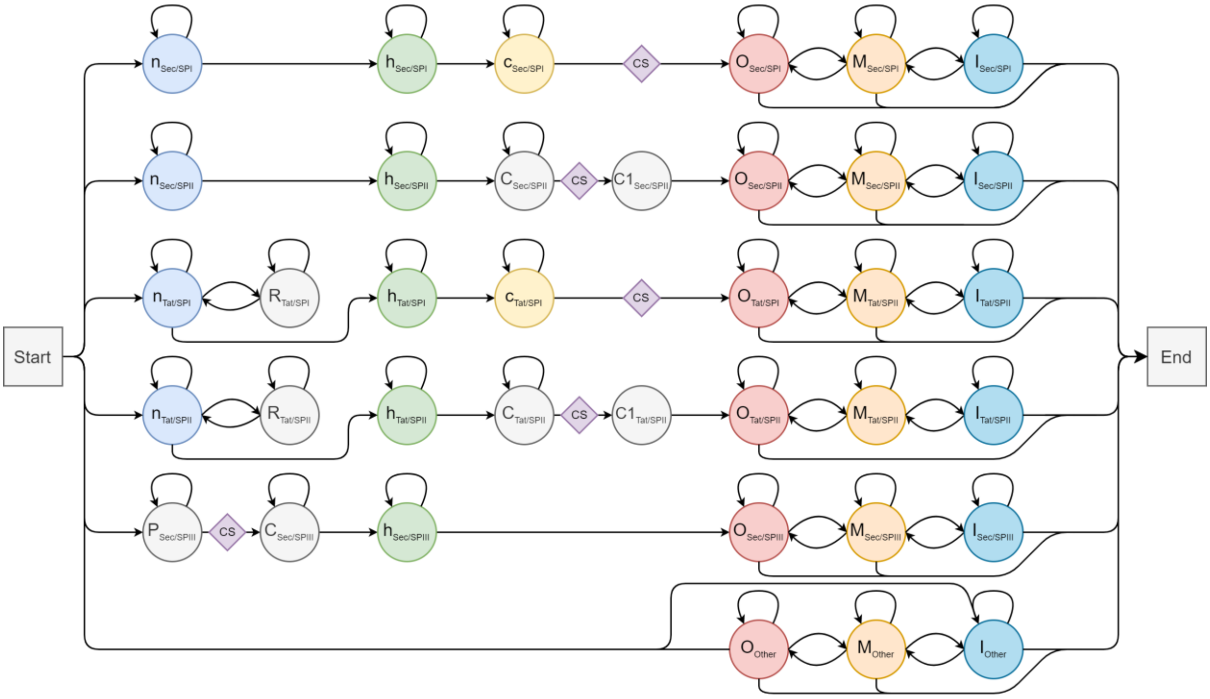

Supplementary Figure 8. States modeled by the CRF. The three regions are indicated by their lower-case name. R is the state of the twin-arginine motif. In lipoproteins, C marks the lipobox and C1 marks the cysteine in +1 of the cleavage site. CS indicates the position of the cleavage site, which is not modeled as a state, but inferred from the end of the previous region. For Sec/SPIII, the whole SP is modeled as a single state P, followed by a conserved and a hydrophobic region. O, M, and I mark extracellular, transmembrane and intracellular regions of the mature protein.

Method	Archaea		Eukarya	Gram-negative bacteria		Gram-positive bacteria	
	MCC1	MCC2	MCC1	MCC1	MCC2	MCC1	MCC2
SignalP 6.0	0.737	**0.728**	**0.868**	0.811	**0.649**	0.878	**0.734**
SignalP 5.0 retrained	0.711	0.67	0.774	0.705	0.586	0.798	0.669
DEEPSIG	n.d.	n.d.	0.792	0.735	0.159	0.798	0.146
LipoP	0.775	0.619	0.347	0.744	0.471	**0.879**	0.442
PHILIUS	0.691	0.438	0.448	0.766	0.147	0.752	0.084
PHOBIUS	0.796	0.551	0.531	0.766	0.153	0.716	0.08
PolyPhobius	0.715	0.474	0.478	0.813	0.173	0.777	0.136
PRED-LIPO	0.733	0.552	0.196	0.71	0.342	0.879	0.484
PRED-SIGNAL	**0.908**	0.67	0.265	0.662	0.115	0.822	0.171
PRED-TAT	0.781	0.655	0.34	0.736	0.209	0.839	0.238
SIGNAL-CF	n.d.	n.d.	0.333	0.52	0.123	0.474	0.1
Signal-3L 2.0	n.d.	n.d.	0.605	0.731	0.108	0.878	0.133
SOSUIsignal	n.d.	n.d.	0.368	0.639	0.123	0.702	0.107
SPEPlip	n.d.	n.d.	0.652	0.705	0.489	0.578	0.429
SPOCTOPUS	0.732	0.448	0.506	**0.849**	0.165	**0.879**	0.134
TOPCONS2	0.711	0.438	0.504	0.844	0.159	0.836	0.078
SignalP 5.0 original	0.899	0.886	0.863	0.821	0.77	0.921	0.868

Supplementary Table 1. Benchmark results for Sec/SPI prediction. MCC1 refers to detection performance when the negative class consists of soluble and transmembrane proteins. For MCC2, the negative class additionally contains Sec/SPII, Tat/SPI and Tat/SPII SPs.

Method	Archaea				Eukarya				Gram-negative bacteria				Gram-positive bacteria			
	±0	±1	±2	±3	±0	±1	±2	±3	±0	±1	±2	±3	±0	±1	±2	±3
CS recall																
SignalP 6.0	0.500	0.556	0.556	0.583	**0.747**	**0.774**	**0.808**	**0.829**	0.639	0.672	0.689	0.721	0.800	0.800	0.800	0.800
SignalP 5.0 retrained	0.389	0.472	0.472	0.528	0.63	0.651	0.705	0.760	0.508	0.574	0.656	0.672	0.733	0.733	0.733	0.733
DEEPSIG	n.d.	n.d.	n.d.	n.d.	0.603	0.63	0.658	0.699	0.508	0.574	0.574	0.574	0.733	0.733	0.800	0.800
LipoP	0.389	0.528	0.556	0.639	0.288	0.329	0.370	0.404	0.656	0.705	0.721	0.721	0.467	0.467	0.533	0.533
PHILIUS	0.500	0.611	0.611	0.611	0.596	0.658	0.712	0.760	0.623	0.672	0.721	0.754	0.467	0.467	0.467	0.467
PHOBIUS	0.472	0.583	0.611	0.639	0.637	0.671	0.699	0.753	0.557	0.656	0.721	0.738	0.467	0.467	0.467	0.467
PolyPhobius	0.528	0.667	0.667	0.667	0.623	0.678	0.733	0.801	0.557	0.672	0.754	0.754	0.667	0.667	0.733	0.733
PRED-LIPO	0.472	0.556	0.611	0.639	0.068	0.082	0.130	0.158	0.410	0.475	0.508	0.525	**0.867**	**0.867**	**0.867**	**0.867**
PRED-SIGNAL	**0.861**	**0.917**	**0.917**	**0.917**	0.226	0.267	0.301	0.329	0.426	0.492	0.607	0.639	0.800	0.800	0.800	0.800
PRED-TAT	0.556	0.694	0.75	0.778	0.370	0.445	0.500	0.548	**0.656**	**0.721**	0.754	0.770	**0.867**	**0.867**	**0.867**	**0.867**
Signal-3L 2.0	n.d.	n.d.	n.d.	n.d.	0.644	0.671	0.719	0.753	0.607	0.639	0.672	0.705	0.733	0.733	0.800	0.800
Signal3Lv2	n.d.	n.d.	n.d.	n.d.	0.664	0.685	0.726	0.753	0.541	0.607	0.623	0.639	0.800	0.800	0.800	0.800
SOSUIsignal	n.d.	n.d.	n.d.	n.d.	0.151	0.308	0.459	0.568	0.246	0.377	0.557	0.623	0.200	0.267	0.267	0.467
SPEPlip	n.d.	n.d.	n.d.	n.d.	0.685	0.712	0.747	0.781	0.574	0.656	0.705	0.721	0.600	0.600	0.667	**0.667**
SPOCTOPUS	0.333	0.389	0.417	0.472	0.384	0.514	0.678	0.747	0.426	0.656	**0.820**	**0.869**	0.600	0.667	0.733	**0.867**
TOPCONS2	0.389	0.528	0.556	0.583	0.329	0.452	0.596	0.692	0.443	0.541	0.656	0.689	0.267	0.333	0.333	0.400
SignalP 5.0 original	0.611	0.694	0.722	0.778	0.692	0.740	0.767	0.815	0.672	0.705	0.738	0.738	0.933	0.933	0.933	0.933
CS precision																
SignalP 6.0	**0.643**	**0.714**	**0.714**	**0.75**	**0.661**	**0.685**	**0.715**	**0.733**	**0.534**	**0.562**	**0.575**	**0.603**	**0.632**	**0.632**	**0.632**	**0.632**
SignalP 5.0 retrained	0.519	0.630	0.630	0.704	0.514	0.531	0.575	0.620	0.378	0.427	0.488	0.500	0.500	0.500	0.500	0.500
DEEPSIG	n.d.	n.d.	n.d.	n.d.	0.587	0.613	0.640	0.680	0.134	0.151	0.151	0.151	0.089	0.089	0.098	0.098
LipoP	0.359	0.487	0.513	0.590	0.141	0.162	0.182	0.199	0.339	0.364	0.373	0.373	0.152	0.152	0.174	0.174
PHILIUS	0.353	0.431	0.431	0.431	0.168	0.186	0.201	0.215	0.110	0.118	0.127	0.133	0.051	0.051	0.051	0.051
PHOBIUS	0.340	0.420	0.440	0.460	0.245	0.258	0.268	0.289	0.099	0.117	0.129	0.132	0.051	0.051	0.051	0.051
PolyPhobius	0.352	0.444	0.444	0.444	0.181	0.197	0.213	0.233	0.098	0.118	0.133	0.133	0.069	0.069	0.076	0.076
PRED-LIPO	0.386	0.455	0.500	0.523	0.052	0.062	0.098	0.119	0.203	0.236	0.252	0.26	0.325	0.325	0.325	0.325
PRED-SIGNAL	0.508	0.541	0.541	0.541	0.073	0.086	0.097	0.106	0.085	0.098	0.121	0.128	0.083	0.083	0.083	0.083
PRED-TAT	0.426	0.532	0.574	0.596	0.08	0.097	0.109	0.119	0.133	0.147	0.153	0.157	0.101	0.101	0.101	0.101
Signal-3L 2.0	n.d.	n.d.	n.d.	n.d.	0.103	0.108	0.115	0.121	0.104	0.110	0.115	0.121	0.067	0.067	0.074	0.074
Signal3Lv2	n.d.	n.d.	n.d.	n.d.	0.357	0.368	0.39	0.404	0.116	0.130	0.134	0.137	0.093	0.093	0.093	0.093
SOSUIsignal	n.d.	n.d.	n.d.	n.d.	0.032	0.066	0.098	0.122	0.042	0.065	0.096	0.107	0.021	0.028	0.028	0.049
SPEPlip	n.d.	n.d.	n.d.	n.d.	0.362	0.377	0.395	0.413	0.278	0.317	0.341	0.349	0.257	0.257	0.286	0.286
SPOCTOPUS	0.240	0.280	0.300	0.340	0.127	0.170	0.224	0.247	0.070	0.107	0.134	0.142	0.062	0.068	0.075	0.089
TOPCONS2	0.275	0.373	0.392	0.412	0.110	0.151	0.199	0.231	0.078	0.095	0.115	0.121	0.029	0.036	0.036	0.043
SignalP 5.0 original	0.647	0.735	0.765	0.824	0.635	0.679	0.704	0.748	0.719	0.754	0.789	0.789	0.824	0.824	0.824	0.824

Supplementary Table 2. Benchmark results for CS prediction in Sec/SPI at different tolerance windows.

Method	Archaea		Gram-negative bacteria		Gram-positive bacteria	
	MCC1	MCC2	MCC1	MCC2	MCC1	MCC2
SignalP 6.0	**0.871**	**0.719**	0.838	0.841	**0.894**	**0.893**
SignalP 5.0 retrained	**0.871**	**0.719**	**0.884**	**0.874**	0.883	0.866
LipoP	**0.871**	0.681	0.806	0.813	0.71	0.724
PRED-LIPO	0.728	0.608	0.615	0.655	0.762	0.743
SPEPlip	n.d.	n.d.	0.856	0.86	0.842	0.837
SignalP 5.0 original	0.937	0.881	0.939	0.925	0.922	0.917

Supplementary Table 3. Benchmark results for Sec/SPII prediction. MCC1 refers to detection performance when the negative class consists of soluble and transmembrane proteins. For MCC2, the negative class additionally contains Sec/SPI, Tat/SPI and Tat/SPII SPs.

Method	Archaea				Gram-negative bacteria				Gram-positive bacteria			
	±0	±1	±2	±3	±0	±1	±2	±3	±0	±1	±2	±3
**CS recall**												
SignalP 6.0	**0.778**	**0.778**	**0.778**	**0.778**	0.852	0.852	0.856	0.864	0.875	0.883	0.883	0.883
SignalP 5.0 retrained	**0.778**	**0.778**	**0.778**	**0.778**	**0.895**	**0.895**	**0.895**	**0.907**	**0.900**	**0.900**	**0.900**	**0.900**
LipoP	**0.778**	**0.778**	**0.778**	**0.778**	0.837	0.837	0.837	0.837	0.700	0.700	0.700	0.700
PRED-LIPO	0.556	0.556	0.556	0.556	0.646	0.646	0.646	0.646	0.767	0.767	0.767	0.767
SPEPlip	n.d.	n.d.	n.d.	n.d.	0.887	0.887	0.891	0.891	0.850	0.850	0.850	0.850
SignalP 5.0 original	0.889	0.889	0.889	0.889	0.949	0.949	0.949	0.953	0.917	0.917	0.917	0.917
**CS precision**												
SignalP 6.0	0.583	0.583	0.583	0.583	0.913	0.913	0.917	0.925	0.929	0.938	0.938	0.938
SignalP 5.0 retrained	0.583	0.583	0.583	0.583	0.895	0.895	0.895	0.907	0.931	0.931	0.931	0.931
LipoP	0.636	0.636	0.636	0.636	0.951	0.951	0.951	0.951	0.955	0.955	0.955	0.955
PRED-LIPO	**0.714**	**0.714**	**0.714**	**0.714**	**0.954**	**0.954**	0.954	0.954	0.939	0.939	0.939	0.939
SPEPlip	n.d.	n.d.	n.d.	n.d.	0.954	0.954	**0.958**	**0.958**	**0.962**	**0.962**	**0.962**	**0.962**
SignalP 5.0 original	0.889	0.889	0.889	0.889	0.953	0.953	0.953	0.957	0.965	0.965	0.965	0.965

Supplementary Table 4. Benchmark results for CS prediction in Sec/SPII at different tolerance windows.

Method	Archaea		Gram-negative bacteria		Gram-positive bacteria	
	MCC1	MCC2	MCC1	MCC2	MCC1	MCC2
SignalP 6.0	0.802	**0.807**	**0.946**	**0.934**	0.788	**0.806**
SignalP 5.0 retrained	0.807	0.763	0.719	0.732	0.708	0.700
PRED-TAT	0.937	0.719	0.945	0.869	**0.823**	0.643
TatP	0.733	0.474	0.730	0.591	0.568	0.411
TATFIND	**0.937**	0.662	0.892	0.845	0.711	0.580
SignalP 5.0 original	0.937	0.719	0.973	0.934	0.931	0.880

Supplementary Table 5. Benchmark results for Tat/SPI prediction. MCC1 refers to detection performance when the negative class consists of soluble and transmembrane proteins. For MCC2, the negative class additionally contains Sec/SPI, Sec/SPII and Tat/SPII SPs.

Method	Archaea				Gram-negative bacteria				Gram-positive bacteria			
	±0	±1	±2	±3	±0	±1	±2	±3	±0	±1	±2	±3
**CS recall**												
SignalP 6.0	**0.333**	**0.444**	0.444	0.444	0.706	**0.765**	**0.784**	0.804	0.556	0.556	**0.667**	0.667
SignalP 5.0 retrained	0.222	0.444	0.444	0.444	0.412	0.451	0.490	0.490	0.167	0.222	0.222	0.278
PRED-TAT	**0.333**	**0.444**	**0.667**	**0.667**	**0.725**	**0.765**	**0.784**	**0.824**	**0.611**	**0.611**	**0.667**	**0.722**
TatP	0.222	0.333	0.444	0.444	0.588	0.608	0.608	0.627	0.333	0.333	0.389	0.389
TATFIND	n.d.	n.d.	n.d.	n.d.	n.d.	n.d.	n.d.	n.d.	n.d.	n.d.	n.d.	n.d.
SignalP 5.0 original	0.333	0.444	0.556	0.556	0.686	0.745	0.784	0.804	0.667	0.667	0.833	0.833
**CS precision**												
SignalP 6.0	**0.375**	**0.500**	**0.500**	**0.500**	**0.679**	**0.736**	**0.755**	**0.774**	**0.714**	**0.714**	**0.857**	**0.857**
SignalP 5.0 retrained	0.182	0.364	0.364	0.364	0.488	0.535	0.581	0.581	0.273	0.364	0.364	0.455
PRED-TAT	0.231	0.308	0.462	0.462	0.638	0.672	0.690	0.724	0.458	0.458	0.500	0.542
TatP	0.133	0.200	0.267	0.267	0.326	0.337	0.337	0.348	0.167	0.167	0.194	0.194
TATFIND	n.d.	n.d.	n.d.	n.d.	n.d.	n.d.	n.d.	n.d.	n.d.	n.d.	n.d.	n.d.
SignalP 5.0 original	0.231	0.308	0.385	0.385	0.660	0.717	0.755	0.774	0.667	0.667	0.833	0.833

Supplementary Table 6. Benchmark results for CS prediction in Tat/SPI at different tolerance windows

Parameter	Net charge	Hydrophobicity
t-statistic	-4.505	3.483
Difference in mean	-0.761	2.978
Confidence interval 95%	-1.096, -0.428	1.283, 4.673
Cohen's d	-0.874	0.674
Degrees of freedom	98.670	103.779
P value	1.821×10^{-5}	7.261×10^{-4}

Supplementary Table 7. Welch's t-test (two-sided) of n-region properties of synthetic SPs, as presented in Supplementary Figure 6.

Species name	Taxonomy ID	Other	Sec/SPI	Sec/SPII	Sec/SPIII	Tat/SPI	Tat/SPII	Note
Methanosaeta harundinacea	1110509	2,065	260	33	0	0	0	Max. Sec/SPI frequency in Archaea
Methanolacinia petrolearia	679926	2,511	163	97	8	0	0	Max. Sec/SPII frequency in Archaea
Halorussus sp. MSC15.2	2283638	3,704	94	24	2	141	107	Max. Tat/SPI frequency in Archaea
Natrialba swarupiae	2448032	3,585	43	11	2	43	133	Max. Tat/SPII frequency in Archaea
Candidate division MSBL1 archaeon SCGC-AAA382A13	1698279	439	6	0	5	0	0	Max. Sec/SPIII frequency in Archaea
Acidilobus sp. SCGC AC-742_M05	1987489	280	1	0	0	0	0	Min. Sec/SPI frequency in Archaea
archaeon HR04	2035440	1,311	50	0	0	1	0	Min. Sec/SPII frequency in Archaea
Methanoplanus limicola DSM 2279	937775	2,666	166	88	8	0	0	Min. Tat/SPI frequency in Archaea
Candidate division TM6 bacterium JCVI TM6SC1	1306947	526	304	24	6	0	0	Max. Sec/SPI frequency in Bacteria
Nannocystis exedens	54	6,725	669	1,664	5	55	25	Max. Sec/SPII frequency in Bacteria
Roseomonas stagni DSM 19981	1123062	4,961	403	109	0	421	8	Max. Tat/SPI frequency in Bacteria
Eggerthella sp. (strain YY7918)	502558	2,476	51	44	5	14	84	Max. Tat/SPII frequency in Bacteria
Victivallis vadensis	172901	3,167	493	184	210	5	0	Max. Sec/SPIII frequency in Bacteria
Buchnera aphidicola (Stegophylla sp.)	2315800	354	0	0	0	0	0	Bacterial endosymbiont
Mycoplasma canadense	29554	426	0	55	0	0	0	Min. Sec/SPI frequency in Bacteria
Candidatus Mikella endobia	1778264	272	1	0	0	0	0	Bacterial endosymbiont, Min. Sec/SPII frequency in Bacteria
Candidatus Termititenax dinenymphae	2218523	335	10	10	0	0	0	Min. Tat/SPI frequency in Bacteria
Escherichia coli (strain K12)	83333	3,854	378	123	8	27	1	
Thermus thermophilus	300852	2,028	132	39	9	18	1	
Deinococcus radiodurans	243230	2,744	238	91	4	7	1	
Corynebacterium glutamicum	196627	2,887	99	94	0	3	10	
Thermotoga maritima	243274	1,706	121	21	4	0	0	
Bacillus subtilis	224308	3,988	151	113	4	4	0	
Archaeoglobus veneficus	693661	1,954	59	41	4	5	2	
Haloferax volcanii	309800	3,699	57	16	3	31	105	
Methanocaldococcus jannaschii	243232	1,717	31	28	11	0	0	
Prometheoarchaeum syntrophicum	2594042	3,813	102	18	0	0	0	
Pyrococcus furiosus	186497	1,937	75	29	4	0	0	

Supplementary Table 8. Selected UniProt reference proteome predictions. Species with no SPs predicted (n=9) were excluded for the determination of bacteria with minimum frequencies.

**Organism group**	**% Sec/SPI**	**% Sec/SPII**	**% Sec/SPIII**	**% Tat/SPI**	**% Tat/SPII**
Archaea (n=330)	2.65 ± 1.96	0.58 ± 0.66	0.12 ± 0.17	0.43 ± 0.62	0.71 ± 1.05
Eukarya (n=1588)	8.08 ± 3.97	-	-	-	-
Gram-positive (n=3387)	3.10 ± 1.43	2.78 ± 1.2	0.11 ± 0.16	0.21 ± 0.29	0.14 ± 0.27
Gram-negative (n=4610)	9.84 ± 3.87	3.98 ± 2.87	0.24 ± 0.38	0.73 ± 0.71	0.24 ± 0.10

Supplementary Table 9. Average frequencies and standard deviations of the five signal peptide types in UniProt reference proteomes.

	Eukarya	Archaea	Gram-positive	Gram-negative
Sec/SPI	2040 (2840)	44 (61)	142 (214)	356 (537)
Sec/SPII	-	12 (27)	516 (782)	1087 (1452)
Sec/SPIII	-	10 (10)	4 (6)	56 (97)
Tat/SPI	-	13 (24)	39 (110)	313 (461)
Tat/SPII	-	6 (6)	8 (11)	19 (19)
Other	14356 (17627)	110 (124)	226 (258)	933 (1077)

Supplementary Table 10. Composition of the SignalP 6.0 training set. Numbers in parentheses are counts before application of the homology partitioning procedure.

	Eukarya	Archaea	Gram-negative bacteria	Gram-positive bacteria
Sec/SPI	146	36	61	15
Sec/SPII	-	9	257	120
Tat/SPI	-	9	51	18
Tat/SPII	-	5	5	3
Other	5581	81	133	81

Supplementary Table 11. Composition of the SignalP 5.0 benchmark dataset after 1) removal of all sequences that were not retained in the new homology partitioning and 2) reclassification of Gram-negative and Tat/SPI samples to Gram-positive and Tat/SPII.

True \ Pred.	Other	Sec/SPI	Sec/SPII	Sec/SPIII	Tat/SPI	Tat/SPII
**Other**	3,393 (3,392)	14 (16)	2 (0)	1 (1)	1 (2)	0 (0)
**Sec/SPI**	12 (8)	1,046 (1,056)	11 (5)	0 (0)	1 (1)	0 (0)
**Sec/SPII**	1 (1)	6 (7)	638 (637)	0 (0)	0 (0)	1 (1)
**Sec/SPIII**	0 (0)	0 (0)	0 (0)	43 (43)	0 (0)	0 (0)
**Tat/SPI**	1 (0)	10 (15)	1 (0)	0 (0)	213 (206)	5 (9)
**Tat/SPII**	0 (0)	0 (0)	0 (0)	0 (0)	0 (0)	3 (3)

Class	MCC 2
**Other**	0.982 (0.989)
**Sec/SPI**	0.987 (0.989)
**Sec/SPII**	0.988 (0.988)
**Sec/SPIII**	0.969 (0.970)
**Tat/SPI**	0.956 (0.957)
**Tat/SPII**	0.577 (0.480)

Supplementary Table 12. Comparison of the distilled model to the full ensemble model, using the set of sequences that were removed by the partitioning procedure as the test set. The performance of the full ensemble model is given in parentheses.
